# High-Performance Liquid Chromatography as a Novel Method for the Determination of α-Defensins in Synovial Fluid for Diagnosis of Orthopedic Infections

**DOI:** 10.3390/diagnostics10010033

**Published:** 2020-01-09

**Authors:** Pavel Melicherčík, Eva Klapková, Karel Kotaška, David Jahoda, Ivan Landor, Václav Čeřovský

**Affiliations:** 1 Department of Orthopaedics, First Faculty of Medicine, Charles University in Prague and Motol University Hospital, V Úvalu 84, 150 06 Prague 5, Czech Republic; pavel.melichercik@fnmotol.cz (P.M.); david.jahoda@post.cz (D.J.); landor@atlas.cz (I.L.); 2 Department of Medical Chemistry and Clinical Biochemistry, Second Faculty of Medicine, Charles University in Prague and Motol University Hospital, V Úvalu 84, 150 06 Prague 5, Czech Republic; eva.klapkova@fnmotol.cz (E.K.); kotaska@email.cz (K.K.); 3 Institute of Organic Chemistry and Biochemistry of the Czech Academy of Sciences, Flemingovo nám. 2, 166 10 Prague 6, Czech Republic

**Keywords:** synovial fluids biomarkers, α-defensins, HPLC, periprosthetic joint infections, infectious arthritis, arthrosis

## Abstract

The α-defensins (AD) present in synovial fluid have been regarded as constituting the most accurate periprosthetic joint infection (PJI) biomarker. The methods most commonly used for estimating AD as a biomarker are the qualitative Synovasure^®^ PJI tests, based on the technique of lateral flow, and quantitative enzyme-linked immunosorbent assay (ELISA). Here, we propose a novel test based on detecting α-defensins in synovial fluid by high-performance liquid chromatography (HPLC). Synovial fluid was collected from 157 patients diagnosed with PJI, infectious arthritis (IA), arthrosis, reactive arthritis, and rheumatoid arthritis. AD concentrations in the fluid were determined by HPLC, and these same samples were used for additional diagnostic analyses. The results were statistically processed to calculate cutoff concentrations for PJI and IA. HPLC testing showed a sensitivity of 94% and a specificity of 92% for diagnosis of PJI, and a sensitivity of 97% and a specificity of 87% for diagnosis of IA. Using HPLC, we detected in synovial fluid a combination of three α-defensins: human neutrophil peptides HNP1, HNP2, and HNP3. All measured AD concentration values shown in this work refer to the sum of these three individual concentrations. Our study shows that the HPLC method meets the conditions for measuring precise concentrations of the sum of AD and can be recommended as part of a diagnostic array for PJI and IA diagnostics. By this method, we have verified that higher levels of AD in synovial fluid can also be seen in rheumatoid illnesses, crystal arthropathies, and reactive arthritis.

## 1. Introduction

Early diagnosis of infectious complications in orthopedics and traumatology is essential, not only for deciding the course of treatment, but also for improving the outcome of the treatment itself. Diagnosis of periprosthetic joint infections (PJI) and infectious arthritis (IA) requires a comprehensive approach. In recent years, the examination of synovial fluid has been recognized as essential for diagnosing PJI [1,2]. The most accurate biomarker in the synovial fluid appears to consist of three human neutrophil peptides (HNP1, HNP2, and HNP3) belonging to the group of human α-defensins [2,3,4,5,6]. Human α-defensins (AD) are active against many Gram-positive and -negative bacteria, certain enveloped viruses, and fungi [6,7,8,9]. HNP1–3 are cationic peptides composed of 29 or 30 amino acids, and each contains three internal disulfide bridges [7,8,9]. They are produced by activated neutrophils in response to various microbial agents or proinflammatory cytokines in the locomotor region and then secreted in synovial fluid [3,10].

For diagnosis of PJI in clinical practice, the long-standing Synovasure^®^ PJI lateral flow test is used as a qualitative in vitro diagnostic tool to confirm or rule out manifestation of this infection [11,12]. The test is based on measured AD concentrations that are either higher or lower than a determined cutoff. The level of AD in synovial fluid also can be determined quantitatively, utilizing enzyme-linked immunosorbent assay (ELISA). ELISA provides results with sensitivity up to 97% and specificity to 100%, even as it is unaffected by systemic inflammatory diseases or by antibiotics use [1,4,5,13,14,15].

The accuracy of AD examination as a PJI biomarker is determined by correct setting of the cutoff concentration. In practice, this means that if the measured AD level is higher than the cutoff concentration, then the synovial fluid is infected. Appropriate cutoff concentration settings are stipulated by accurate diagnoses from a patient population. In the case of PJI, the Musculoskeletal Infection Society (MSIS) criteria seem to comprise the most objective set of cutoff levels [2,16,17,18]. For the lateral flow method, the cutoff for AD concentration for PJI is set to 5.2 mg/L [4,5,10].

When determining AD concentration in joint fluid as a PJI diagnostic marker, it is important to note that a test result could be a false positive or false negative. Therefore, debate has recently arisen over whether AD as a diagnostic biomarker measured by the lateral flow method is indeed sufficiently explicit for the accurate diagnosis of PJI.

In our institutions, we have invented a new method for determining precise concentrations of the AD in synovial fluid that is based upon high-performance liquid chromatography (HPLC). In this context, the main objective of the present work was to determine the AD cutoff concentrations (counted as the sum of HNP1, HNP2, and HNP3) for the cases of PJI and IA using this HPLC method. Furthermore, in the comparison with the two techniques commonly used to date, the Synovasure lateral flow device (Synovasure^®^ PJI test) and the ELISA test, we discuss various viewpoints on using the HPLC method for AD determination as a biomarker. We then discuss the use of these tests’ outputs in clinical practice.

## 2. Materials and Methods

### 2.1. Patients

Our study enrolled 157 patients of different age categories and sexes. Based on the medical history, clinical examination, laboratory tests, and imaging methods for each, we divided the patients into six groups: I. PJI (18 patients, 16 knee, 2 hip), II. noninfectious patients with total endoprosthesis (26 patients, 24 knee, 2 hip), III. infectious arthritis (34 patients), IV. arthrosis (31 patients), V. reactive arthritis with crystalline arthropathies (19 patients), and VI. rheumatoid arthritis (29 patients). The study was approved by the Ethics Committee for Multi-Centric Clinical Trials of the Motol University Hospital (Reference No: EK-132/15, approval date 4 February 2015. Informed consent was given by all patients prior to the trial and before the retrospective evaluation.

We collected the patients’ synovial fluid by puncture (in groups III–VI only from the knee) and mixed it (exactly measured volume of 1 mL) with a stabilizing solution of 1:1 acetonitrile/water mixture containing 0.5% trifluoroacetic acid (4 mL). This strongly acidic aqueous and organic solvent mixture provides optimum solubility and stability for the cationic AD peptides. This was done under sterile conditions, primarily at the outpatient clinic but also during surgery. The study was conducted from May 2015 to March 2016.

To diagnose arthritis, we used anamnestic data, clinical and X-ray examinations, laboratory blood tests such as for C-reactive proteins (CRP), erythrocyte sedimentation rate (ESR), rheumatic factor (RF), uric acid, and antinuclear antibodies (ANA), and synovial fluid analysis (culture, polymerase chain reaction (PCR), leukocyte esterase, and AD). Patients with PJI as well as the noninfectious patients with total endoprosthesis were diagnosed based on two major criteria of the Musculoskeletal Infection Society (MSIS): (1) a sinus tract communicating with the prosthesis, and (2) a pathogen isolated by culture from the fluid as well as from the tissue [17]. All aseptic patients (TEP) had negative cultures.

### 2.2. HPLC Analysis

HNP1 ≥98% (Cat. No. SRP3126) as a standard, acetonitrile and trifluoroacetic acid were obtained from Sigma-Aldrich, Prague, Czech Republic.

The calibration curve of HNP-1 was constructed by measuring seven calibration standard concentrations: 2, 5, 10, 20, 30, 50, and 100 mg/L. Two levels of control samples were prepared at concentrations of 25 and 50 mg/L. Quantification was based on the peak area. The analytical method has been successfully validated. The calibration was linear across the whole range of concentrations with mean correlation coefficients *R*^2^ of 0.99713. The intra- and interday accuracy and precision were evaluated on two QC samples by multiple analysis (*n* = 20). The intra-assay CVs were 4.2% and 3.5%. The interassay CVs were 5.4%, and 4.1%. The within-day accuracy expressed by the calculated bias between observed and theoretical concentrations for albumin was 1.8% and 1.7%. The limit of quantification was found to be 2.0 mg/L. The first point of calibration curves (2.0 μg/mL) corresponds to the lower limit of quantification (LLOQ).

**Figure 1:**
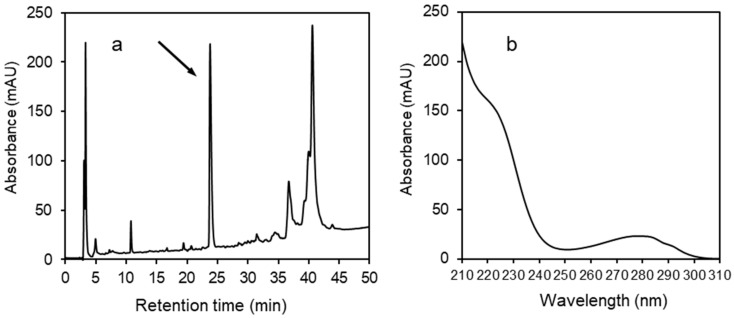
(**a**) An example of an RP-HPLC profile of joint fluid at 220 nm. The arrow indicates the peak representing the combination of three human α-defensins (HNP-1, HNP-2, and HNP-3). (**b**) The characteristic UV spectrum of α-defensins.

The procedure for HPLC analysis was as follows: An aliquot of the synovial fluid in stabilizing solution was further diluted with acetonitrile (1:1). This sample was then analyzed by reversed‑phase high‑performance liquid chromatography (RP-HPLC) to quantify the concentration of α-defensins (HNP1–3). RP-HPLC was carried out on an Agilent 1260 Infinity system (Agilent Technologies, Santa Clara, CA, USA) equipped with a diode array detector, quaternary pump system, column thermostat, auto sampler injecting a volume of 50 μL, and a Vydac 218 TP C18, 250 × 4.6 mm, 5 μm, column (Grace Vydac, Hesperia, CA, USA). We used a solvent gradient ranging from 5% to 70% acetonitrile/water/0.1% trifluoroacetic acid at a 1 mL/min flow rate over 60 min at 22 °C. The elution was monitored by absorption at 220 nm utilizing a diode-array detector. The instrument was controlled using OpenLab Software (Santa Clara, CA, USA). The quantity of AD was calculated from its peak area at 220 nm based on a comparison with the peak area of a standard solution of HNP1. The selected fraction (peak corresponding to α-defensins, Figure 1) was collected, the solvent was evaporated in a Speed-Vac (Labconco, Kansas City, MO, USA), and the material was analyzed by electrospray ionization mass spectrometry (ESI-MS) in the service department of the institute. 

### 2.3. Data Analysis

Values of *p* < 0.05 were considered statistically significant. A D’Agostino–Pearson normality test was used to determine the normality of the data distribution. Receiver operating characteristic analysis was used to investigate the diagnostic efficiency. Cochran’s Q test and Cohen’s kappa statistic test were used to evaluate the diagnostic validity of α-defensins, microbial cultivation, and PCR in distinguishing between the infectious and noninfectious origins of orthopedic diseases. The statistical software GraphPad Prism, version 8.01 (San Diego, CA, USA), and MedCalc software, version 18.02.01 (Oostende, Belgium), were used.

## 3. Results

A selected example of an RP-HPLC profile at 220 nm for one of the synovial fluids is shown in Figure 1a. The components eluted in the peak at 24.7 min were identified by electrospray ionization mass spectrometry (ESI-MS) as the combination of three human α‑defensins with molecular masses of 3439.53 for HNP1, 3368.49 for HNP2, and 3483.50 for HNP3 (Figure S1). They exhibit the characteristic UV spectrum shown in Figure 1b. The size of peak areas varied according to the extent and type of disease and differed dramatically from patient to patient. Measured values for AD (HNP1–3) concentrations (mg/L) obtained from 157 patients of different diagnoses are shown in Table S1.

**Figure 2:**
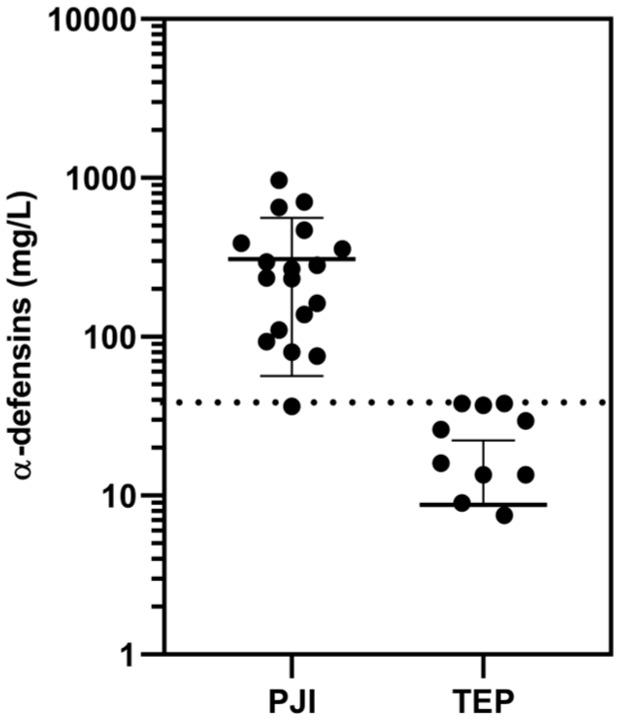
Synovial fluid α-defensins concentrations (logarithmic scale) for the patients with periprosthetic joint infections (PJI) versus those with aseptic total endoprosthesis (TEP), as determined by HPLC. The cutoff concentration (38 mg/mL) is represented by the dotted line.

Based on these values and our statistical analysis, we determined the cutoff concentrations of AD (the sum of HNP1–3) for PJI and IA. The cutoff concentration of AD for PJI was 38 mg/L (AUC = 0.99; sensitivity: 94%, 95% CI: 0.73–1; specificity: 92%, 95% CI: 0.75–0.99) when comparing the results for patients with PJI against the patients with total endoprosthesis (TEP), who are regarded as aseptic. As can be seen in Figure 2, the group of patients with PJI shows only one false negative result.

**Figure 3:**
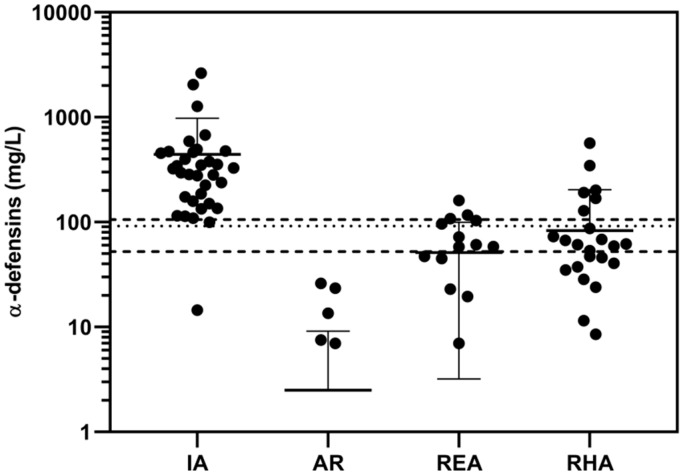
Synovial fluid α-defensins concentrations (logarithmic scale) for the patients with infectious arthritis (IA) versus patients with aseptic diseases: arthrosis (AR), reactive arthritis (REA), and rheumatoid arthritis (RHA). The cutoff concentration (98 mg/mL) is represented by the dotted line. The two broken lines enclose what can be termed a “gray zone” within the range 63–108 mg/L. The lower broken line also indicates the cutoff concentration (62.5 mg/mL) in the case of comparison of IA, but only against AR.

For infectious arthritis (IA), the cutoff concentration of AD was determined to be 62.5 mg/L (AUC = 0.998; sensitivity: 97%, 95% CI: 0.85–0.98; specificity: 100%, 95% CI: 0.89–1.00) when comparing the results of patients with IA against aseptic patients with arthrosis (AR). See Figure 3. The group of patients with IA shows only one false negative result.

In addition, this figure also shows the α-defensins concentrations of patients with IA compared to the α-defensins concentrations measured for three groups of patients with arthrosis (AR), reactive arthritis (REA), and rheumatoid arthritis (RHA) (these three groups of patients were considered aseptic).

In this case, the cutoff concentration of AD for IA was determined to be 98 mg/L (AUC = 0.95; sensitivity: 97%, 95% CI: 0.85–1; specificity: 87%, 95% CI: 0.78–0.94).

Due to the higher AD concentrations obtained in some patients diagnosed with noninfectious reactive arthritis and rheumatoid arthritis, we have determined what might be termed a “gray zone” around the cutoff values in the range of 63–108 mg/L (Figure 3).

In the group of 24 patients with different diagnoses we compared the validity of the determination of AD concentration obtained by the HPLC method with those for the joint fluid examination by PCR and cultivation (Table 1).

We used Cochran’s Q test to evaluate the validity of those methods used (HPLC, microbial cultivation, and PCR) in determining the infectious or noninfectious origin of the disease. Cochran’s test showed significant differences (*p* < 0.05) between the results obtained in determining AD by HPLC, PCR, and cultivation. That means the validity and diagnostic power of those methods used also differed. Using Cohen’s kappa statistic to evaluate which methods had significant diagnostic validity, we found AD determination using the HPLC method to be the most significant. The weighted kappa (κ) value for HPLC was 0.91, showing almost perfect diagnostic power. PCR followed with a κ value of 0.76, showing very good compliance, and then analysis by cultivation methods with a κ value of 0.41, showing good compliance.

## 4. Discussion

Synovial AD are the most accurate biomarkers currently used in the diagnosis of PJI [6,10,17,18]. In this work, we have shown that AD concentration in synovial fluid can easily be determined by HPLC technique. Unlike the other commercially available tests discussed below, HPLC can be used not only for determining PJI, but also for diagnosing IA and other arthropathies. In our hospital, we already have introduced this method into the repertoire of standard examinations for infectious joint diseases.

The technique most commonly used today for determination of AD from synovial fluid in PJI is commercially available under the name Synovasure^®^ PJI test. This qualitative test is based on the method of immunochromatographic lateral flow [11,12,19]. The first studies of this method were highly optimistic, with the results of those examinations showed specificity and sensitivity exceeding 90% [11,12].

The test seemed much more beneficial than the more commonly used screening methods (cultivation, histology, PCR, leukocyte esterase, and others). Recently, however, debate has arisen over this method’s accuracy [14,20,21]. This concern has been focused primarily on the results of studies that questioned the benefit of determining AD from synovial fluid, where, in particular, sensitivity was lower and varied in comparison with previous results. For the lateral flow method, sensitivity is between 67% and 100% and specificity 94% to 97% [11,12,16,21].

Very good accuracy of the Synovasure^®^ PJI test had been reported by Gehrke et al. for diagnosis of PJI after total hip arthroplasty or total knee arthroplasty [12]. When considering data from a wider range of patients, the overall sensitivity of the test was 92.1% and specificity was 100% [12]. Berger et al. also reported that Synovasure^®^ PJI test had an excellent diagnostic performance, showing sensitivity of 97.1% and specificity of 96.6% [11]. As they pointed out, however, this test can only confirm or refute the diagnosis of PJI. Nevertheless, the test should be considered an important part of a diagnostic set of biomarkers for PJI. On the other hand, Synovasure^®^ PJI test has not yet been used for differential diagnosis of arthritis.

Because examination for the presence of AD as a diagnostic marker in joint fluid for PJI by the Synovasure^®^ PJI test does not have 100% sensitivity and specificity, we have encountered both false positive and negative results in our clinical practice, similarly as described in the literature. In our practice, we also have noticed that the results of the test are not reliable in cases of acute PJI, especially due to the occurrence of false negative findings. False negative results of the Synovasure^®^ lateral flow method in PJI may be also due to the occurrence of low‑virulence bacterial strains (e.g., *Staphylococcus epidermidis* and *Propionibacterium acnes*), as reported by Scholten et al. [21]. In their work, five of 37 patients were diagnosed with PJI based on the intraoperative tissue cultures, but the Synovasure^®^ test confirmed the infection in only one of these five cases.

The false positive results obtained by determining AD level in PJI diagnosis may occur in patients with crystal arthropathies [22], as well as in cases of rheumatoid arthritis and reactive arthritis [3,23]. Another possible cause of elevated AD levels in joint fluid can be a presence of metallosis around the joint replacement [13,19].

The lateral flow method of the Synovasure^®^ test offers a rapid perioperative examination within a few minutes, provided that the concentration of hemoglobin in synovial fluid does not exceed 0.5 g/dL, as stated in the Synovasure^®^ PJI booklet. In spite of these limiting factors, the lateral flow method is a very useful diagnostic part of PJI diagnosis. This test offers very useful and important information during follow-up examinations after joint replacements, providing results within minutes and without the need for laboratory equipment, albeit at a high cost per test.

Compared to the qualitative measurement of AD with the Synovasure^®^ lateral flow device, it is apparent that the quantitative measurement of AD concentrations in synovial fluid by the ELISA method has greater predictive value for diagnosing PJI. For example, the combination of synovial fluid AD measured by ELISA method and CRP test has demonstrated sensitivity of 97% and specificity of 100% for diagnosis of PJI [4]. Some additional, detailed studies dealing with the testing of synovial AD by ELISA have been published (e.g., in the work of Deirmengian et al. [1,5], Bonanzinga et al. [13], and Ahmad et al. [24]), with all reporting sensitivity and specificity of the test in the range of 97–100%. As reported by Ahmad et al. from a study based on meta-analysis of synovial biomarkers in PJI, the measurement of AD by ELISA in several respects outperforms that of the Synovasure^®^ lateral flow device [24]. On the other hand, it does take longer to evaluate the results.

In this work, we present an entirely new method based on RP-HPLC. It enables quantifying the exact concentration of the sum of three human AD in joint fluid, specifically that of HNP1, HNP2, and HNP3. Because these three peptides notably differ in their sequence only in one amino acid residue at their N-terminus [9], this chromatographic technique does not allow for their separation and they accordingly elute together in a single narrow peak (Figure 1a). Because the amino acid tryptophan occurs in their sequences, HNP1–3 exhibit high absorbance in the ultraviolet light (UV) range and have a characteristic UV spectrum (Figure 1b). This facilitates easy and unequivocal detection of the defensins peak during the chromatography. Moreover, the occurrence of each particular defensin within the HPLC peak can be further confirmed by mass spectrometry (Figure S1), as we did in the case related to Figure 1.

In the departments of our hospital, we have used HPLC to measure precise AD concentrations not only for determining PJI, but also for IA diagnoses. On the basis of our measurements, we verified that AD secretion into synovial fluid is also increased in those patients affected with rheumatic diseases and other arthropathies due to an etiology different than that for the infectious type. These patients were confirmed in this work as aseptic. For these reasons, the cutoff concentration for IA was estimated to be 98 mg/L (Figure 3), which is higher compared to the cutoff concentration for PJI (38 mg/L). For IA, therefore, we have set the diagnostic window as a gray zone in the range of 63–108 mg/L (Figure 3). On the other hand, the cutoff concentration for IA was 62.5 mg/mL when comparing the AD concentrations against the concentrations taken only from one group of aseptic patients with AR (Figure 3). Alpha-defensins can thus be produced in increased or reduced amounts and in varying proportions of their subtypes individually in patients diagnosed with PJI and IA.

Based on our experience, the most accurate diagnosis of PJI and IA results from the combination of synovial fluid examination by PCR, microbial culture cultivation, and HNP1–3 determination by HPLC (Table 1). Despite the fact that the microbial cultures had the least significant validity (κ = 0.402), this examination is still very important, especially for the determination of the susceptibility of infectious agents to antibiotics. The PCR method was (in our particular cases) hampered by false positivity from the contamination and false negativity from an unrepresentative sample. Nevertheless, the benefit of this examination was quite high (κ = 0.755). Using HPLC we could relatively easily obtain the exact concentrations of AD in the synovial fluid. Although this is an indirect determination of infection and inflammation in the joint, its diagnostic benefit is significant (κ = 0.905). Together with the results of other examinations, we were able to responsibly decide whether we were dealing with PJI, IA, or another disorder (Table 1).

HPLC, above all, provides accurate measurements of AD concentrations, thereby allowing better interpretation of the results obtained vis-à-vis other screening methods. Because the testing takes somewhat longer, examination by HPLC seems to be more suitable for diagnosing outpatients. Another benefit of using HPLC is that the results are not affected by the presence of blood in the sample, and the method can also be used for IA diagnosis. 

As we have proven here, AD are present in the synovial fluid as a mixture of the three peptides HNP1, HNP2, and HNP3. Under the chromatography conditions that we used, however, it was not feasible to separately quantify each of these peptides in the mixture. Although the HPLC method used in our laboratory is based on determining the sum of AD, the ratio of their individual amounts within the mixture does not affect the accuracy of the diagnosis. A reasonable question, of course, is whether the proportion of individual AD subtypes within the mixture varies depending on the diagnosis or extent of the disease. Provided that the conditions for the separation of individual defensins by the chromatographic technique are reasonable, this might be an interesting topic for future research.

Synovial fluid AD seem to be the most reliable biomarkers that could be used in a perioperative test for the diagnosis of PJI. While to date the qualitative lateral flow test and the quantitative ELISA test for AD determination have been used, to our knowledge, and regrettably, only a few comparative studies about their performance have been reported [12,15,24]. It must also be stressed that none of those studies is concerned about the proportion of individual α-defensins in the synovial fluid samples. Both types of tests are commercially available as kits. A comparison of various viewpoints on the use of individual screening methods, including HPLC, for the determination of synovial α-defensins concentration as a biomarker is shown in Table 2.

We conducted this study on patients with six types of diagnosis (PJI, TEP, IA, AR, REA, and RHA). Thus, it may appear that comparing α-defensin concentrations between these groups may be inappropriate. However, we demonstrated that by measuring the α-defensin concentrations by HPLC, the diagnosis of the patients from the PJI group was clearly distinguished from the diagnosis of the TEP group. Also, the diagnosis of the patients from the IA group was clearly distinguished from the AR group. In addition, we detected elevated α-defensin levels in several REA and RHA patients. As these concentrations were not high compared to the IA group, we included them in the noninfectious etiology group. Based on the AUC values, which are higher than 0.90 for all measured combinations, and the values of specificities and sensitivities, we can unambiguously confirm the diagnostic significance of our determination by HPLC.

## 5. Conclusions

To our knowledge, this is the first study dealing with the use of HPLC for assessing the concentration of α-defensins in joint fluid as a biomarker. Unlike the other two tests discussed above, the HPLC method provides direct measurements of the sum of AD concentration expressed in mg/L. In our hospital, we verified that this method, in combination with other standard clinical examinations, contributed significantly not only to the confirmation or exclusion of the PJI and IA diagnoses, but also to a quantitative estimate of disease extension. Provided that the orthopedic surgeons have an appropriately outfitted testing facility at hand, advantages also include the fact that the results of the HPLC test can be provided within 1 h and at minimal cost.

## Figures and Tables

**Table 1:** Synovial fluid examination for α-defensins (HNP1–3) by HPLC, microbial culture cultivation, and PCR, in patients with orthopedic diseases of infectious and noninfectious origin.

Patient	Cultivation	PCR	AD by HPLC	Diagnosis
1	neg.	pos.	pos.	IA
2	neg.	pos.	pos.	IA
3	neg.	pos.	pos.	IA
4	neg.	neg.	pos.	IA
5	neg.	pos.	pos.	IA
6	neg.	pos.	pos.	IA
7	pos.	pos.	pos.	PJI
8	neg.	pos.	pos.	PJI
9	pos.	pos.	pos.	PJI
10	neg.	pos.	pos.	PJI
11	neg.	pos.	pos.	PJI
12	neg.	pos.	pos.	PJI
13	neg.	neg.	pos.	PJI
14	neg.	neg.	neg.	TEP *
15	neg.	pos.	neg.	TEP *
16	neg.	neg.	neg.	TEP *
17	neg.	neg.	neg.	TEP *
18	neg.	neg.	neg.	TEP *
19	neg.	neg.	pos.	REA *
20	neg.	neg.	neg.	REA *
21	neg.	neg.	neg.	RHA *
22	neg.	neg.	neg.	AR *
23	neg.	neg.	neg.	AR *
24	neg.	neg.	neg.	AR *

AR = arthrosis, IA = infectious arthritis, neg. = negative test result, PJI = periprosthetic joint infection, pos. = positive test result, REA = reactive arthritis, RHA = rheumatoid arthritis, TEP = total endoprosthesis. A positive test in the case of AD means a finding greater than the determined cutoff concentration. In the cases of cultivation and PCR, positive means a presence of bacteria was determined. A negative test in the case of AD means a finding less than the determined cutoff concentration. In the cases of cultivation and PCR, negative means an absence of bacteria was determined. Weighted kappa (κ) values showing the diagnostic validity of the methods used with respect to the determination of infections were as follow: HPLC: κ = 0.905, PCR: κ = 0.755, microbial cultivation: κ = 0.402. The diagnostic validity of AD measured by HPLC is most significant. The noninfectious diseases are marked with an asterisk (*).

**Table 2:** Comparison from various viewpoints of methods available for detecting α-defensins in synovial fluid to diagnose periprosthetic joint infections (PJI) and infectious arthritis (IA).

Viewpoint	Synovasure^®^ PJI Test (Lateral Flow Device)	ELISA	HPLC
α-defensins	Sum of HNP1–3	HNP1, HNP3, or sum of HNP1–3	Sum of HNP1–3
Type of examination	Diagnostic device	Laboratory exam using diagnostic kit	Laboratory exam using instrument
Test output	Presence/absence of PJI	Exact concentration of single α-defensin	Exact concentration of the sum of α-defensins
Time to result	Within 10 min	Within hours	Within 1 h
Cost	Expensive	Less expensive	Low cost
Cutoff concentration	5.2 mg/L ^a^, 7.72 mg/L ^b^ only for PJI	5.2 mg/L ^c^ only for PJI	38 mg/L for PJI 98 mg/L for IA
Blood in sample	If hemoglobin exceeds 0.5 g/dL, result is affected	Does not affect result	Does not affect result ^d^

^a^ [4,5,10], ^b^ [10], ^c^ [4,5,10,16], ^d^ we did not identify the presence of HNP1–3 in noninfectious samples with a high blood content.

## Supplementary Materials

The following are available online at https://www.mdpi.com/2075-4418/10/1/33/s1, Figure S1: Electrospray ionization mass spectrometry (ESI-MS) spectra of the human α-defensins, HNP1, HNP2, and HNP3. Table S1: α-Defensins (HNP1–3) concentrations obtained by HPLC measurements in patients with various diagnoses.
