# Supplementary material for: High-Performance Liquid Chromatography as a Novel Method for the Determination of α-Defensins in Synovial Fluid for Diagnosis of Orthopedic Infections
Source: Diagnostics (Basel). 2020 Jan 9;10(1):33. doi: 10.3390/diagnostics10010033 (PMC7167866; doi:10.3390/diagnostics10010033)
Supplement: Supplementary file 1 [file diagnostics-10-00033-s001.pdf]

Figure S1: Electrospray ionization mass spectrometry (ESI-MS) spectra of the human α-defensins, HNP1, HNP2, and HNP3.

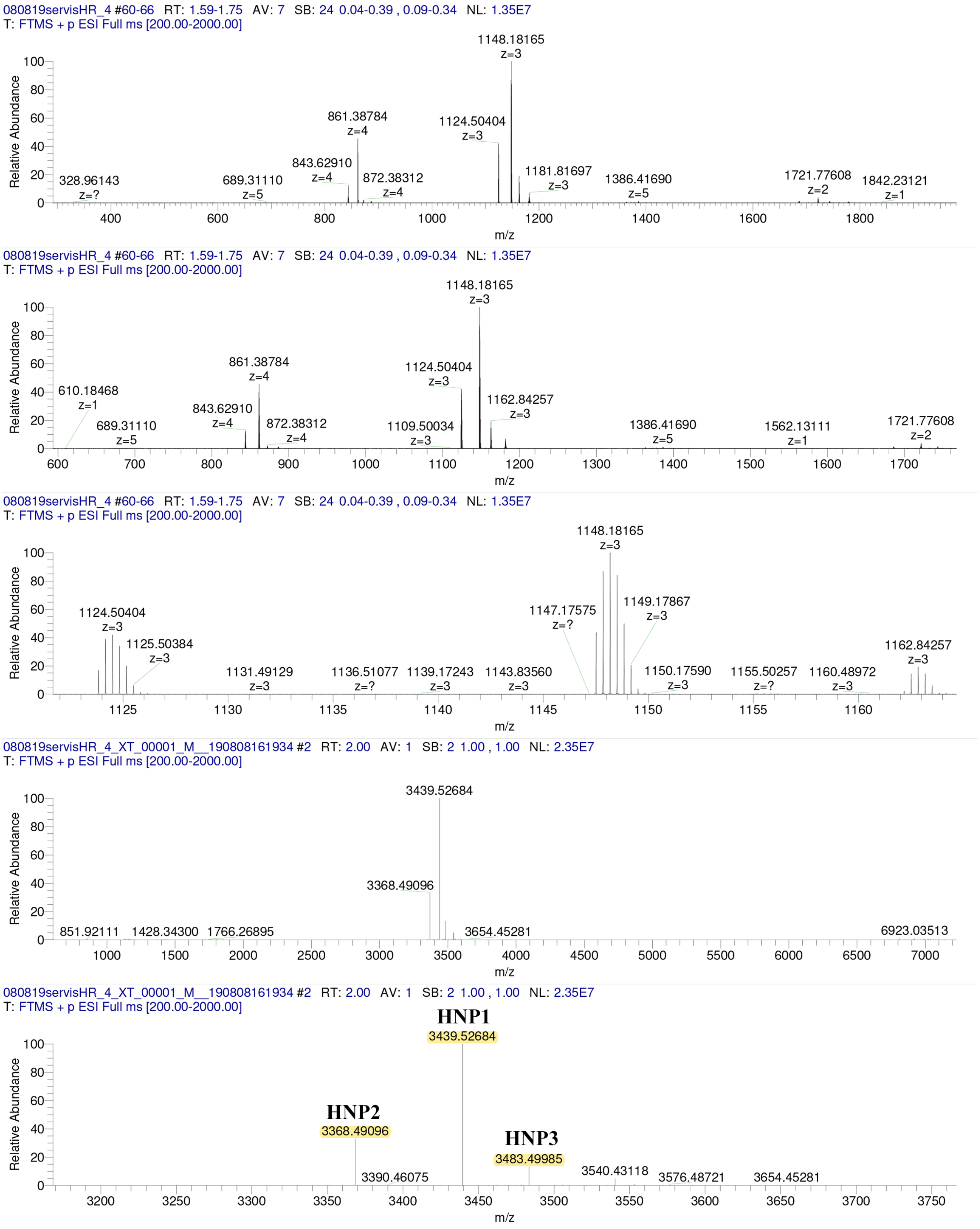

Table S1: α-Defensins (HNP1-3) concentrations obtained by HPLC measurements in patients with various diagnoses.

Patient	α-defensin (HNP1-3) concentrations (mg/L)					
	**PJI**	**TEP**	**IA**	**AR**	**REA**	**RHA**
1	234.5	38	115	0.0	0.0	28.5
2	232	0.0	150	0.0	45	0.0
3	356	0.0	99.5	0.0	0.0	201
4	75.5	0.0	14.5	0.0	0.0	0.0
5	80	13.5	2042	0.0	47	0.0
6	36.5	37	677.5	0.0	0.0	0.0
7	653	0.0	133.5	0.0	61	0.0
8	388.5	0.0	328	0.0	23	37.4
9	965.5	13.5	186	0.0	116.5	191.5
10	468.5	0.0	174	13.5	96	73
11	295.5	29.5	454	7.5	19.5	67
12	110	38	109	26	103.5	59
13	93	16	135,5	0.0	7	68.5
14	138.0	0.0	113,5	0.0	58	345.5
15	162.5	0.0	1272	0.0	0.0	128.5
16	267	0.0	2635	0.0	160.5	0.0
17	282	0.0	277	0.0	108	47
18	706	0.0	323.5	23.5	58.5	168.5
19		0.0	348.5	0.0	72	53.5
20		0.0	377.5	0.0		11.5
21		7,5	470.5	0.0		46
22		0.0	477	0.0		87
23		0.0	238.5	0.0		35
24		26.0	284.5	0.0		40.5
25		0.0	591.5	0.0		8.5
26		9	464.5	0.0		61
27			343.5	7.0		24
28			398.5	0.0		62
29			282.5	0.0		565.5
30			158	0.0		
31			355,5	0.0		
32			225,0			
33			493			
34			296			

AR = arthrosis, IA = infectious arthritis, PJI = periprosthetic joint infections, REA = reactive arthritis, RHA = rheumatoid arthritis, TEP = total endoprosthesis.
